# Walking the Straight and Narrow: The Moderating Effect of Evaluation Apprehension on the Relationship between Collectivism and Corruption

**DOI:** 10.1371/journal.pone.0123859

**Published:** 2015-03-27

**Authors:** Zhen-wei Huang, Li Liu, Wen-wen Zheng, Xu-yun Tan, Xian Zhao

**Affiliations:** 1 School of Psychology, Beijing Normal University, Beijing, China; 2 Department of Psychology, University of Kansas, Lawrence, Kansas, United States of America; Mälardalen University, SWEDEN

## Abstract

Previous studies obtained mixed results regarding the association between collectivism and corruption. To make sense of the mixed results, the current research examined the moderating role of evaluation apprehension on the relationship between collectivism and corruption. Study 1, using a bribery scenario, indicated that collectivism facilitated corruption only when evaluation apprehension was low. Study 2, using a real money bribery game, confirmed the moderated model found in Study 1. Study 3 further demonstrated the different effects of vertical/horizontal collectivism on corruption. Our results suggest that a society may effectively combat corruption by increasing its social costs while, at the same time, retaining its collectivistic values.

## Introduction

Sociologists, economists and psychologists have attempted to establish a connection between collectivism and corruption [1–3]. While it seems that we should not preserve collectivistic values to walk the straight and narrow, what is the alternative? We argue that evaluation apprehension can aid corruption prevention for collectivism. Evaluation apprehension can influence attitudes and behaviors in general [4, 5], and impede corruption in particular [6]. Previous studies on the association between collectivism and corruption paid little attention to evaluation apprehension. Thus, the overall purpose of the current research is to examine the moderating role of evaluation apprehension on the association between collectivism and corruption.

### Can Collectivism Promote Corruption?

Collectivism is the extent to which individuals see themselves as an interdependent part of a larger group or society [2, 7]. There is remarkable consensus that collectivism facilitates corruption [8–10]. Cross-national analyses also show that collectivism is positively related to bribe perception [11, 12] and corruption perception [2]. Findings at the individual level of analysis further demonstrate a positive association between collectivism and corruption [1–3, 13, 14]. However, there are some exceptions in country-level analysis. The positive impact of collectivism on corruption is dampened when per capita income or other culture dimension is included in the model [15, 16]. Furthermore, Bernardi, Delorey [6] found that the acceptability of corruption increases as a country’s collectivism decreases. The reason for this inconsistent finding remains unknown.

### Evaluation Apprehension, Collectivism and Corruption

Evaluation apprehension has an effect on corruption. Evaluation apprehension is an active, anxiety-toned concern that one receives a positive evaluation from others, or at least that one provides no grounds for a negative evaluation [5]. As such, evaluation apprehension can be manipulated by certain subtle social cues. For instance, the presence of an audience, an imaged audience [17] or even an eye-like picture could elevate evaluation apprehension [17–21]. Psychologists have long known about the problem of evaluation apprehension in self-report measures. Behaviors in some domains are more evaluative than others [22]. Corruption is one such behavior. As there is notable agreement that corruption is a morally repugnant business practice [2, 23], one may receive a negative evaluation if he/she engages in corrupt behavior. Therefore, evaluation apprehension can hamper corruption. Consistent with this, Bernardi, Delorey [6] find a negative effect of evaluation apprehension on corruption.

It is argued that the inconsistent findings regarding the association between collectivism and corruption may be due to differences in evaluation apprehension. Accumulating evidence suggests people high in collectivism are more likely to have higher evaluation apprehension compared to people low in collectivism [24–26]. For example, it has been demonstrated that evaluation apprehension has effects on service satisfaction when a collectivistic self-construal is primed, whereas evaluation apprehension exerts little impact on service satisfaction when an individualistic self-construal is primed [4]. Accordingly, we predict that collectivism is positively related to corruption when evaluation apprehension is low, but it is negatively related to corruption when evaluation apprehension is high (Hypothesis 1).

### Evaluation Apprehension, Vertical/Horizontal Collectivism and Corruption

Triandis et al. proposed to include horizontal (valuing equality) and vertical (emphasizing hierarchy and competition) aspects in the analysis of individualism-collectivism [27, 28]. Thus one can distinguish two types of collectivism: horizontal collectivism (HC) and vertical collectivism (VC). People with a horizontal collectivism orientation are especially focused on sociability and on treating others with benevolence and loyalty. In contrast, people with a vertical collectivism orientation are particularly focused on dutifully fulfilling their obligations to others [28].

The horizontal–vertical dimension adds a nuance to the understanding of the relationship between collectivism and corruption. Li, Triandis [1] demonstrate that horizontal and vertical collectivism have different effects on corruption such that vertical collectivism is positively related to corruption, while horizontal collectivism is not. The evaluation apprehension may explain these different effects. A primary concern associated with horizontal collectivism is to save face and maintain good relationships with others [24, 29, 30], thus implying that people high in horizontal collectivism would be more concerned about evaluation than would people low in horizontal collectivism. Along similar lines, Lalwani, Shavitt [24] find that horizontal collectivism significantly and positively predicts evaluation apprehension, while vertical collectivism does not. It is thus reasonable to argue that evaluation apprehension restrains people high in horizontal collectivism from engaging in corruption, but it does not exert any effect for people high in vertical collectivism. Based on the foregoing analysis, we predict that 1) horizontal collectivism is negatively related to corruption when faced with high evaluation apprehension, but positively related to corruption when faced with low evaluation apprehension (Hypothesis 2a) and that 2) high vertical collectivism individuals are more likely to engage in corruption compared to low vertical collectivism individuals, whether evaluation apprehension is high or low (Hypothesis 2b).

### Overview of Studies

The present research examined the possible effect of collectivism on corruption by varying social contexts in which corruption were elicited. As the most common type of corruption is bribery [15, 16, 31–33], corruption was operationalized as bribery in the present research. Study 1 aimed to test hypothesis 1. In this study, collectivism was measured with a scale, corruption was measured by the reactions to a bribery scenario, and the manipulation of evaluation apprehension was embedded in the same scenario. Study 2 sought to replicate the results of Study 1 by using the same collectivism scale, a different behavioral measurement of corruption (real money bribery games) and a different subtle cue of social evaluation (an eye-like picture). Study 3 aimed to test hypotheses 2a and 2b. In this study, horizontal and vertical collectivism were measured with another scale, corruption was measured by a bribery game, and the manipulation of evaluation apprehension was the same as in Study 2.

## Study 1

### Methods

#### Ethics Statement

The study was reviewed and approved by the Committee of Protection of Subjects at Beijing Normal University. All participants provided written informed consent before the study, and they were fully debriefed at the end of the research according to the established guidelines of the committee. This procedure was followed in Study 2 and Study 3 as well.

#### Participants

One hundred and sixteen students (100 females and 16 males; mean age = 20.9 years, *SD* = 1.62) in introductory courses at two large universities in Beijing participated in the study. The participants were randomly assigned to the evaluation apprehension condition (*N* = 60) or the neutral condition (*N* = 56) in a between-participants design.

#### Materials

Collectivism and individualism. The Singelis [34] scale was used to measure collectivism, α = .75 (12 items; e.g., “It is important for me to maintain harmony within my group”), and individualism, α = .69 (12 items; e.g., “My personal identity independent of others is very important to me”). The participants were instructed to indicate the extent to which they endorsed each statement on a 7-point Likert scale (1 = strongly disagree; 7 = strongly agree).

Bribery scenario. We exposed the participants to a bribery scenario in which they assumed the role of a sales agent who had competed against two other firms to win a contract from an international buyer and earn a commission. The sales agent was contemplating whether to offer an unofficial payment to the potential international buyer to help win this contract. The bribery scenario was adapted from Mazar and Aggarwal [2]. After reading, the participants were instructed to complete a 7-point Likert type corruption propensity questionnaire (6 items; e. g., “I think I will give money to him”; α = .89).

The manipulation of evaluation apprehension. In the neutral condition, the bribery scenario was exactly the same in Mazar and Aggarwal [2]. In the evaluation apprehension condition, we added a paragraph revealing the possibility that other people would know what the sales agent did. To check the effect of the manipulation, the participants were asked, “If I offer unofficial payments to the buyer, it is likely that other people would know my conduct”. To determine that our manipulation of evaluation apprehension did not influence the perceived penalty, the participants were asked, “If I offer unofficial payments to the buyer, I would be punished for my conduct”. Responses were again reported on 7-point scales (1 = strongly disagree; 7 = strongly agree).

#### Procedure

All the participants completed the questionnaire in sequential order, which included the following three parts: a collectivism and individualism measure, a bribery scenario, and demographic questions including gender, age, and exposure to corruption. The participants anonymously completed the questionnaires in classrooms.

### Results and Discussion

To confirm the effects of evaluation apprehension manipulation, evaluation apprehension check scores and perceived penalty check scores were analyzed in a one-way (condition: evaluation apprehension vs. neutral) between subjects MANOVA. The main effect of condition on evaluation apprehension check was significant (*M*_evaluation apprehension_ = 5.61, *M*_neutral_ = 5.11), *F*(1, 113) = 4.20, *p* < .05, η^2^ = .04. In contrast, the main effect of condition on perceived penalty was not significant (*M*_evaluation apprehension_ = 4.58, *M*_neutral_ = 4.29), *F*(1, 113) = 0.79, *p* > .05. Thus, it can be concluded that the scenario successfully manipulated the participants’ evaluation apprehension, but not the perceived penalty.

To test the moderating effect of evaluation apprehension, general linear model (GLM) analyses were used. Collectivism and individualism scores were mean centered by subtracting their means from all observations to reduce multicollinearity concerns [35]. Initial analyses were then conducted with these mean-centered variables, and the cross-product of collectivism/individualism with evaluation apprehension provided the interaction term for the model. We recoded gender and evaluation apprehension using contrast coding (male = −1, female = 1; neutral condition = −1, evaluation apprehension condition = 1).

**Fig 1:**
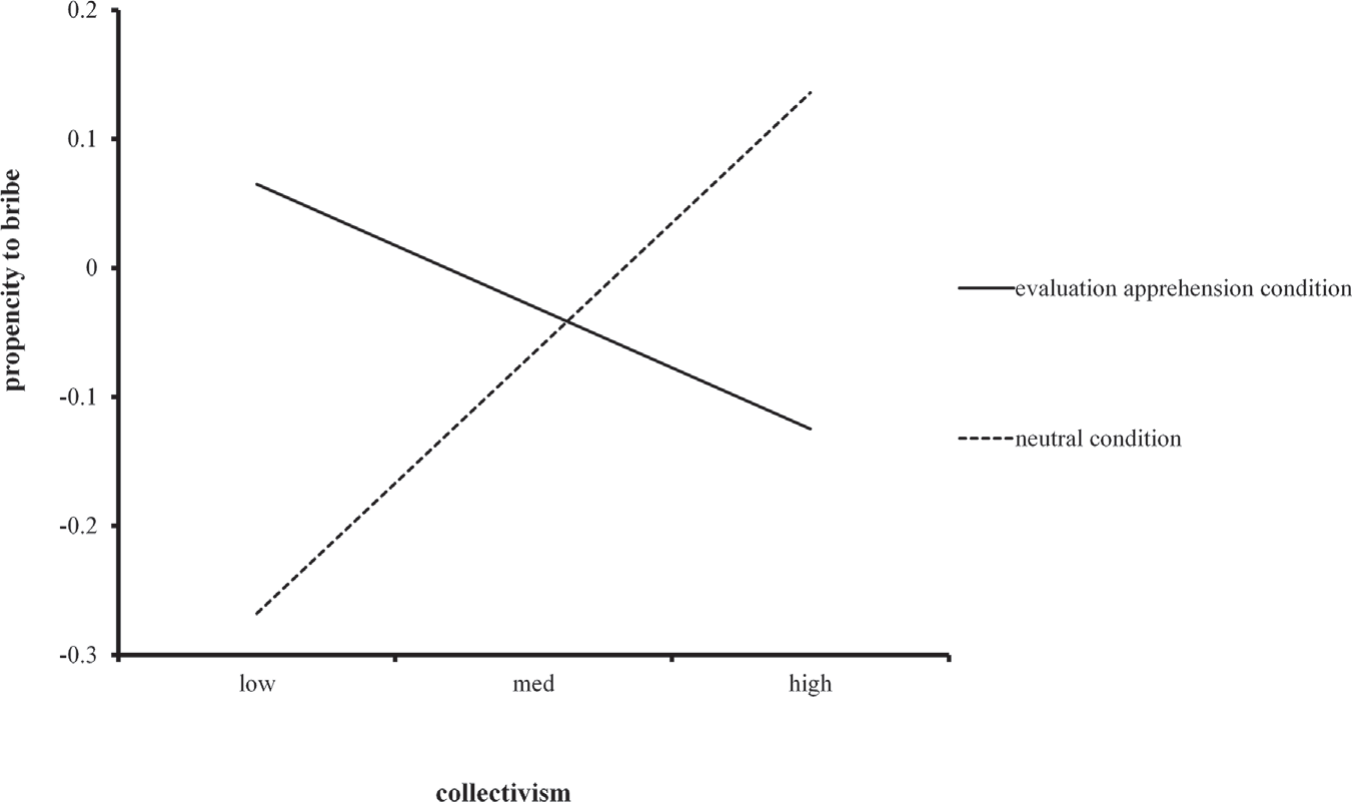
Evaluation apprehension moderates the relationship between collectivism and corruption.

Propensity to bribe scores were then submitted to a condition (neutral vs. evaluation apprehension) × collectivism multiple regression analysis. The collectivism main effect did not attain conventional levels of significance (*B* = 0.03), *t*(112) = 0.12, *p* = .90, and the condition main effect was not significant (*B* = 0.06), *t*(112) = 0.39, *p* = .69. The predicted condition × collectivism effect was significant (*B* = −.62), *t*(112) = −2.96, *p* < .01, Δ*R* ^2^ = 0.075. We further examined this interaction using simple slope analyses [35]. Fig 1 shows the slope of collectivism in the neutral condition and in the evaluation apprehension condition. Our results suggest that when the possibility of being evaluated is low, people with high collectivism are more likely to engage in corruption (*B* = 0.64), *t*(112) = 2.01, *p* < .05; in contrast, when the possibility of being evaluated is high, people with high collectivism are less likely to engage in corruption (*B* = −0.59), *t*(112) = −2.21, *p* < .05. The same analyses were conducted again after including the demographic variables of age, gender and exposure to corruption in separate analyses of covariance, but these did not affect the results. Age (*B* = 0.20, *t*(109) = 2.20, *p* < .05) and exposure (*B* = 0.21, *t*(109) = 2.41, *p* < .05) were significant predictors, while gender was far from significant (*B* = -0.01, *t*(109) = -.01, *p* = .99). Thus, controlling for a variety of demographic factors, the relations between evaluation apprehension, collectivism and corruption remained unchanged. Thus, the results confirm hypothesis 1.

The study suggested that evaluation apprehension moderated the link between collectivism and corruption. Our findings help explain results of previous studies that found mixed and conflicting results regarding the relationship between collectivism and corruption [1–3, 6, 8, 13, 15].

Propensity to bribe scores were then submitted to a condition (neutral vs. evaluation apprehension) × individualism multiple regression analysis. Neither the condition main effect (B = 0.02), *t*(112) = 0.13, *p* = .90, nor the individualism main effect (*B* = .277), *t*(112) = 1.3, *p* = .19, nor the condition × individualism interaction (*B* = .052), *t*(112) = 0.30, *p* = .76 attained conventional levels of significance.

Thus we did not find that evaluation apprehension moderates the relationship between individualism and corruption. It is understandable that we did not get the effects for the individualism scale as the collectivism and individualism scales from the Singelis’s measure are orthogonal [36]. This result is consistent with previous results that collectivism is associated with social evaluation concerns, while individualism is not [24–26]. Accordingly, individualism is not considered in Study 2 and Study 3.

## Study 2

The objective of Study 2 was twofold. Firstly, we sought to ascertain the generalizability of the moderated model found in Study 1 with a different manipulation of evaluation apprehension. In Study 1, evaluation apprehension was manipulated by a scenario. In Study 2, we used an eye-like picture to induce evaluation apprehension. This procedure hides the purpose of the manipulation from participants. Secondly, we sought to further improve the ecological validity of the moderated model using a real money game. In Study 1, corruption was measured by self-report. However, psychologists have long questioned the validity of self-report measures of social behavior [37]. To overcome this methodological limitation, the experiment includes a bribery game with behavioral outcomes for Study 2. In this bribery game, participants could bribe his/her “counterpart” with real money in order to earn a higher payoff.

### Methods

#### Participants

Forty eight participants (26 females and 22 males; mean age = 22.3 years, *SD* = 2.24) participated in the study. Participants received ￥0 to ￥56 (depending on each participant’s performance in the bribery game) as an incentive for their participation. Half of the participants were randomly allocated to the evaluation apprehension condition, and the other half were assigned to the neutral condition.

#### Materials

Collectivism. The same 12-item scale [34] as in Study 1 was used to measure collectivism, α = .69.

Bribery game. A real money bribery game adopted from Abbink and Hennig-Schmidt [38] was used to measure corruption. We adapted the bribery game in two ways so that we could afford it. First, we divided all the amount by 1000, using ￥1 instead of ￥1000. Second, the game was played only once. There were two roles in the game: a firm and a public official. As a potential briber, the firm wished to run an industrial plant that would cause negative consequences to the public. The public official must decide whether to give the firm a permission to operate. The firm, however, could make a private payment to the official in the hopes of influencing the official’s decision. Although we made participants realize that they would be randomly designated as either the firm or the public official, we actually assigned all of the participants to the role of firm. Participants were told that the game was for real money. They made the actual choice between offering and not offering a bribe. If one decided to offer a bribe, one must specify the amount to be sent, which could be an integer of the range from RMB ￥1 to ￥9. They were also told that the use of the ID numbers throughout the experiment would ensure their anonymity.

**Fig 2:**
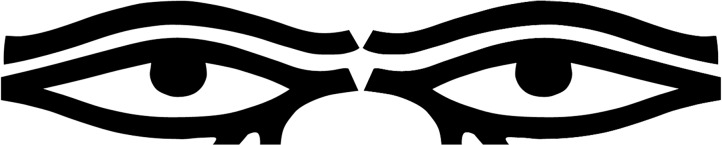
The eye-like picture.

Manipulation of evaluation apprehension. An eye-like picture (the eyes of Horus) adopted from Haley and Fessler [20] was used to prime evaluation apprehension (Fig 2). A flower image adopted from Bateson, Nettle [18] was used for the neutral condition. To check the effect of the manipulation, a 3-item scale adopted from Govern and Marsch [39] were used, α = .64 (e.g., “I am concerned about the way I present myself”).

The eyes of Horus were used to prime evaluation apprehension in Study 2 and 3.

#### Procedure

After accessing the collectivism scale via pencil and paper, participants completed the bribery game, manipulation check, demographic questions and suspicion check via a computer terminal. The eye-like picture or the flower image was displayed at the top of the computer screen until the start of the suspicion check.

### Results and Discussion

The suspicion check showed that no participant correctly guessed the purpose of the study. As expected, participants reported greater evaluation apprehension in the evaluation condition (*M* = 4.66, *SD* = 1.14) than in the neutral condition (*M* = 3.81, *SD* = 1.31), *F*(1, 46) = 5.81, *p* < .05, η^2^ = .11. Analyses were conducted with mean-centered variable, and the cross-product of collectivism with evaluation apprehension provided the interaction term for the model. We recoded gender and evaluation apprehension using contrast coding (male = −1, female = 1; neutral condition = −1, evaluation apprehension condition = 1).

When predicting the dichotomous scoring of bribery, we used a logistic regression analysis. The predictors in this analysis included two sets of variables (main-effect terms and interaction terms) entered in a hierarchical fashion. Neither the main-effect of collectivism (*B* = -0.48, *χ* ^*2*^ (1, *N* = 48) = 0.17, *p* = .62) nor evaluation apprehension (*B* = 0.08, *χ* ^*2*^ (1, *N* = 48) = 0.05, *p* = .82) were significant. The evaluation apprehension × collectivism was significantly related to offering a bribe, *B* = -3.59, *χ* ^*2*^ (1, *N* = 48) = 9.71, *p* < .05. This pattern held with the control variables, including age, gender and exposure to corruption. In addition, age is the only covariates that was marginally significantly related to offering bribery, *B* = .34, *χ* ^*2*^ (1, *N* = 48) = 2.86, *p* = .09.

To further test the moderating effect of evaluation apprehension, we used a linear regression framework, regressing the bribe size on collectivism, evaluation apprehension and the interaction between them. The collectivism main effect did not attain conventional levels of significance (*B* = 0.05), *t*(44) = 0.08, *p* = .94. The condition main effect was not significant (*B* = 0.33), *t*(44) = 1.08, *p* = .28. The predicted condition × collectivism effect was significant (*B* = -2.57), *t*(44) = -3.91, *p* < .001, Δ*R* ^2^ = 0.252. Simple slope analyses show that in the neutral condition, participants with high collectivism offered more money (*B* = 2.62), *t*(44) = 2.66, *p* < .05; in contrast, in the evaluation apprehension condition, participants with high collectivism offered less money (*B* = -2.52), *t*(44) = -2.89, *p* < .01. Finally, the results were the same with the control variables. Age is the only control covariate that was marginally significantly related to briber size, *B* = .39, *t*(41) = 1.68, *p* = .07.

These results support the moderated model found in study 1. Collectivism could facilitate corruption under low evaluation apprehension, but impede corruption under high evaluation apprehension. We demonstrated the replicability of the results of study 1. Study 2 also addresses two weaknesses of Study 1. First, rather than relying on participants to admit to corruption, study 2 uses a game in order to allow participants engage in actual bribery behavior with real money. Second, study 2 uses a subtle social cue to prime evaluation apprehension. We found that even a subtle social cue could evoke evaluation apprehension and exert an influence on corruption.

## Study 3

Study 3 aimed to test hypotheses 2a (evaluation apprehension could moderate the association between horizontal collectivism and corruption) and hypotheses 2b (the moderator does not apply to the association between vertical collectivism and corruption). It was designed to answer the following two questions: Do horizontal and vertical collectivism associate to corruption differently? What is the role of evaluation apprehension plays on these associations?

### Methods

#### Participants

Sixty two participants (42 females and 20 males; mean age = 21.2 years, *SD* = 2.60) participated in the study. They were recruited through a poster displayed at an Internet forum of a university. Participants received forum credits (depending on each participant’s performance in the bribery game) as an incentive for their participation. Half of the participants were randomly allocated to the evaluation apprehension condition, and the other half were assigned to the neutral condition.

#### Materials

Horizontal/Vertical collectivism. An 8-item scale adopted from Triandis and Gelfand [28] was used to measure horizontal and vertical collectivism. For example, “If a coworker gets a prize, I would feel proud” (horizontal collectivism), and “Parents and children must stay together as much as possible” (vertical collectivism). The participants were instructed to indicate the extent to which they endorsed each statement on a 7-point Likert scale (1 = strongly disagree; 7 = strongly agree). The subscale reliabilities (Cronbach’s alpha) were as follows: horizontal collectivism, α = .81; vertical collectivism, α = .64.

Bribery game. A four-shot bribery game adopted from Abbink and Hennig-Schmidt [38] was used to measure corruption. The game began with an instruction [38] that made the participants realize that their payment for participating in the study would be dependent on their performance in the game. There were 4-round games between different players. Thus, the short-term relationships between a briber and a public official were modeled. In each round, participants made the actual choice between offering and not offering a bribe. If one decided to offer a bribe, one must specify the amount to be sent, which could be an integer of the range from RMB ￥1000 to 9000. The subjects were paid anonymously, at an exchange rate of 0.05 forum credits per Yuan. They were also told that the use of the ID numbers throughout the experiment would ensure their anonymity.

Manipulation of evaluation apprehension. The same eye-like picture and flower image as in Study 2 was used to prime evaluation apprehension. We used the same manipulation check [39] as in Study 2, α = .77 (e.g., “I am concerned about the way I present myself”).

#### Procedure

All data were collected using Qualtrics Research Suite. The participants completed the horizontal and vertical collectivism measures, bribery game, manipulation check, demographic questions and suspicion check in sequential order online. After the collectivism measure, the eye-like picture or the flower image was displayed at the top of the computer screen until the start of the suspicion check.

### Results and Discussion

The suspicion check showed that no participant correctly guessed the purpose of the study. Participants in the evaluation apprehension condition reported higher evaluation apprehension (*M* = 4.17, *SD* = 1.74) than did participants in the neutral condition (*M* = 3.34, *SD* = 1.22), *F*(1, 60) = 4.71, *p* < .05, η^2^ = .07, thus suggesting that our manipulation was effective.

To examine the effects of the horizontal collectivism, vertical collectivism evaluation apprehension and any interactions on payments of bribes and sizes of bribes, HLM analyses were conducted. All metric variables were centered such that the intercepts represented the effect for an average person. Table 1 shows the results.

**Table 1:** Predicting Corruption with Horizontal Collectivism, Vertical Collectivism, and Evaluation Apprehension.

HLM level	Model 1		Model 2	
	Bribe payment	Bribe size	Bribe payment	Bribe size
Gender	-.58	-.15	-.42	-.03
Age	.00	.17	-.05	.12
Exposure	-.40*	-.67***	-.25	-.52*
HC	-.09	-.01		
VC			-.63*	.65*
EA	-.25	-.42†	-.28	-.49†
HC × EA	-.71*	-1.16**		
VC × EA			-.22	-.26
Time	-.05	-.12	-.05	-.12

For time effects, approximate *df* s = 240. For all other effects, approximate *df*s = 55. VC = vertical collectivism; HC = horizontal collectivism; EA = evaluation apprehension. Bribe payment was recoded using dummy coding (Not bribe = 0, bribe = 1). Bribe size was divided by 1000. Time was recoded as follows: first time as “1”, second time as “2”, and so on. Gender and evaluation apprehension were recorded using contrast coding (male = −1, female = 1; neutral condition = −1, evaluation apprehension condition = 1).

†*p* < .08.

*p < .05.

**p < 0.01.

***p < 0.001.

Models 1 and 2 show that vertical collectivism significantly predicted corruption, but horizontal collectivism did not. Further, and as expected, horizontal collectivism × evaluation apprehension interaction was significant, but vertical collectivism × evaluation apprehension interaction was not. To probe horizontal collectivism × evaluation apprehension interactions, the simple slopes for neutral condition and evaluation apprehension condition were computed [40]. Horizontal collectivism was positively related to corruption in the neutral condition (bribe payment *B* = .88, *t*(55) = 1.87, *p* = .06; bribe size *B* = 1.15, *t*(55) = 2.60, *p* < .01), but negatively related to corruption in the evaluation apprehension condition (bribe payment *B* = -1.19, *t*(55) = -1.77, *p* < .08; bribe size *B* = -1.16, *t*(55) = -2.36, *p* < .05).

These results support Hypotheses 2a and 2b. Specifically, people high in horizontal collectivism would bribe only in a low evaluation apprehension condition. However, people high in vertical collectivism would bribe in both high and low evaluation apprehension conditions. These results were consistent regardless of how corruption was operationalized (as bribe payment or bribe size).

## General discussion

### Collectivism and Corruption

In sum, we found that collectivism predicted corruption differently depending on evaluation apprehension. We observed a positive association between collectivism and corruption in the neutral condition, and a negative association between collectivism and corruption in the evaluation apprehension condition. We found evidence for this moderated model in Study 1 and 2 in which different measures of bribery and different manipulation of evaluation apprehension were used. These findings help explain when collectivism increases corruption, demonstrating that evaluation apprehension is a crucial influence on this relationship.

Our results indicate that evaluation apprehension helps to understand the mixed results of the relationship between collectivism and corruption. Several researchers argue that collectivism facilitates corruption [1–3, 9, 13]. However, some studies show that there is little association between collectivism and corruption [15, 16]. Furthermore, Bernardi, Delorey [6] originally included evaluation apprehension as a part of their research design and found a surprising negative relationship between collectivism and corruption. To make sense of these mixed results, the current research reveals that evaluation apprehension serves as a boundary condition for the positive association between the two.

However, it is unclear whether evaluation apprehension is high or low in most previous studies exploring the relationship between collectivism and corruption. For instance, Wated and Sanchez [14] electronically mailed or faxed the survey to all enterprises in the sampling frame requesting their participation in the study. Further, most previous researchers did not report the social contexts in which participants completed the measures [1, 2, 13, 41]. According to our results, subtle social cues can evoke evaluation apprehension that moderate relationship between collectivism and corruption. It is hard to make sense of the relationship between collectivism and corruption without consideration of its social context. Future researchers in the field should be cautious about the social cues related to evaluation apprehension in their studies.

It is argued that evaluation apprehension is a variate rather than a covariate. Bernardi, Delorey [6] note that evaluation apprehension should be controlled for in corruption research. However, our results support the notion that evaluation apprehension is a key variable in corrupt research, but not a noise. The difference in evaluation apprehension reflects the different ways people high and low in collectivism pursue their goals. Ignoring the variance of evaluation apprehension or excluding it with statistical control may generate results with little significance of collectivism. The results also support the idea that people high in collectivism are concerned about external evaluation [24–26, 42]. Thus, the importance of the interplay of collectivism and evaluation apprehension must be taken into consideration in the field.

### Different Effects of Vertical and Horizontal Collectivism on Corruption

Study 3 further demonstrated the different effects of vertical/horizontal collectivism on corruption. In Study 3, we found that the relationship between horizontal collectivism and corruption is moderated by evaluation apprehension, but the moderator does not apply to the association between vertical collectivism and corruption, albeit vertical collectivism alone is associated with corruption. The results further advance the findings by Li, Triandis [1] in which vertical collectivism positively predicts corruption, whereas horizontal collectivism does not predict it. The subtle relationships between horizontal collectivism, vertical collectivism, corruption and evaluation apprehension suggest that evaluation apprehension explains the different effects of horizontal and vertical collectivism on corruption.

In addition, our findings add to a growing literature suggesting that the horizontal and vertical distinction enriches our understanding of collectivism. Singelis, Triandis [27] suggest that vertical collectivism is the essential element of collectivism. However, as Lalwani, Shavitt [24] and our results show that it is horizontal collectivism that is similar to collectivism, it is imperative to further investigate the conceptual relations between horizontal collectivism, vertical collectivism and collectivism.

### Practical Implications

Many corrective policies assume that all it takes to curb corruption is to increase its economic costs, for example, increasing the penalty [2]. However, the current research draws attention to the social costs of corruption and demonstrates that some social cues related to evaluation apprehension may be enough to reduce corruption for collectivism in general, and for horizontal collectivism for particular. Moreover, Study 1 demonstrates that evaluation apprehension has nothing to do with economic penalties.

Social scientists often lay the blame on collectivism when they attempt to determine the reason why certain nations have a greater propensity to engage in corruption [1–3]. However, to reduce corruption, “traditional” societies with higher corruption may not need to emulate the societies that have well-grounded “modern” values [43].Several Pacific communities have adopted new institutions to lower corruption while, at the same time, retaining their traditional institutions and values [44]. For example, Samoa formalizes traditional leadership in various kinds of national councils of chiefs. The reports did not find evidence of corruption among these organizations [45]. Our results suggest that a society may effectively combat corruption by increasing its social costs while, at the same time, retaining its collectivistic values.

## Supporting Information

S1 Dataset The data for Study 1. (SAV)

S2 Dataset The data for Study 2. (SAV)

S3 Dataset The level 1 data for Study 3. (SAV)

S4 Dataset The level 2 data for Study 3. (SAV)
